# Comparative real-world study of apalutamide and darolutamide in Japanese patients with non-metastatic castration-resistant prostate cancer

**DOI:** 10.1186/s12894-025-01919-z

**Published:** 2025-09-26

**Authors:** Maiko Ikeda, Koichi Uemura, Yusuke Ito, Hiroki Ito, Takashi Kawahara, Hisashi Hasumi, Jun-Ichi Teranishi, Kazuhide Makiyama, Hiroji Uemura

**Affiliations:** 1 Department of Urology, Yokohama City University Graduate School of Medicine, 3-9, Fukuura, Kanazawa-Ku, Yokohama, Kanagawa 2360004 Japan; 2 Department of Urology and Renal Transplantation, Yokohama City University Medical Center, Yokohama, Japan; 3 Department of Urology, Shonankamakura General Hospital, Kamakura, Japan

**Keywords:** Apalutamide, Castration-resistant, Darolutamide, Prostatic neoplasms, Treatment outcome

## Abstract

**Background:**

Non-metastatic castration-resistant prostate cancer (nmCRPC) is often asymptomatic but carries a risk of progression to metastatic disease. Apalutamide (APA) and darolutamide (DARO) have been shown to improve metastasis-free survival (MFS). This study evaluated the real-world efficacy and safety of APA and DARO in Japanese patients with nmCRPC.

**Methods:**

We retrospectively analyzed 67 nmCRPC patients treated with APA (*n* = 32) or DARO (*n* = 35). Outcomes included time to treatment discontinuation or mCRPC progression, time to mCRPC, PSA response rate, treatment-related adverse events (TRAEs), post-mCRPC treatment patterns, and predictors of progression.

**Results:**

In patients with prostate-specific antigen doubling time (PSADT) < 10 months, no significant difference was observed between the APA and DARO groups in the time to progression to mCRPC. PSA response and MFS were comparable between groups. TRAEs were significantly more frequent with APA (75.0% vs. 25.7%), with rash being the most common. High PSA at treatment initiation (≥ 3.6 ng/mL) and PSA response < 90% were independent predictors of progression. Abiraterone was the most common first-line agent after mCRPC.

**Conclusions:**

DARO was associated with a lower incidence of TRAEs compared to APA. Rash was more prevalent with APA. Elevated baseline PSA and suboptimal PSA response were associated with progression.

**Supplementary Information:**

The online version contains supplementary material available at 10.1186/s12894-025-01919-z.

## Background

Non-metastatic castration-resistant prostate cancer (nmCRPC) is defined as increasing prostate-specific antigen (PSA) levels despite castration levels of serum testosterone and no distant metastases on conventional imaging during androgen deprivation therapy (ADT) or combined androgen blockade (CAB) therapy. Although patients with nmCRPC are often asymptomatic, a significant proportion of patients eventually develop metastatic disease, which is associated with worse clinical outcomes and shorter overall survival (OS).

Androgen receptor pathway inhibitors (ARPIs), such as apalutamide (APA) and darolutamide (DARO), improved metastasis-free survival (MFS) and OS in patients with nmCRPC compared with placebo in the SPARTAN and ARAMIS trials [1–4]. Several studies have reported real-world data on these agents in Japanese patients. When selecting treatment for nmCRPC, not only efficacy and safety but also individualized risk factors must be considered. Understanding the predictors of progression to metastatic CRPC (mCRPC) can help refine treatment strategies and optimize patient outcomes.

In this study, we aimed to evaluate the real-world efficacy and safety of APA and DARO in Japanese patients with nmCRPC. Furthermore, we explored potential predictors of progression to mCRPC and examined treatment patterns following disease progression.

## Methods

### Study design and patient

This was a retrospective study conducted at Yokohama City University Hospital. We included all consecutive patients diagnosed with nmCRPC between 2019 and 2023 at our institution who were treated with APA or DARO. No patients were excluded during this period. Metastatic CRPC (mCRPC) was defined according to the Prostate Cancer Working Group 3 (PCWG3) criteria, including the appearance of two or more new lesions on bone scintigraphy for bone metastasis. PSA progression was defined as a confirmed rise in PSA level of ≥ 25% and ≥ 2 ng/mL above the nadir. All patients had received androgen deprivation therapy (ADT) plus bicalutamide prior to developing CRPC. Prior use of flutamide following bicalutamide and before initiation of APA or DARO was allowed in this analysis.

### Treatment and follow-up

Patients received APA (240 mg/day) or DARO (600 mg/day) as standard initial doses, with no patients starting at a reduced dose. Dose reductions or treatment discontinuation were determined at the discretion of the attending physician. Follow-up evaluations included PSA measurements and imaging studies (computed tomography and bone scintigraphy) every 6–12 months or when PSA levels increased. Treatment sequences were allowed based on clinical judgment. If treatment was switched to another agent due to PSA progression without radiological metastases, patients were still classified under nmCRPC.

### Outcomes

We initially evaluated the time to discontinuation of APA or DARO or progression to mCRPC. This endpoint was defined as the earliest occurrence of any of the following: evidence of stopping the initial APA or DARO treatment, switching to another ARPI, diagnosis of mCRPC, or death. The time to progression to mCRPC was evaluated based on PSADT (< 10 months vs. ≥ 10 months) and compared between the APA and DARO groups. The PSA response rate was defined as the percentage of nadir PSA relative to baseline PSA at the start of APA or DARO treatment. Safety was assessed by comparing the incidence of treatment-related adverse events (TRAEs) between the two treatment groups. TRAEs were graded using the Common Terminology Criteria for Adverse Events (CTCAE) version 5.0. Other outcomes included the location of metastasis, treatment patterns after progression to mCRPC, and predictors of progression to mCRPC.

### Statistical analysis

Group comparisons were performed using the Student’s t-test or chi-square test, as appropriate. Kaplan–Meier analysis and log-rank tests were used to evaluate time-to-event outcomes. Multivariate Cox regression analysis was performed to identify predictors of progression to mCRPC.

Cut-off values for age, time to CRPC, and PSA at treatment initiation were set based on the median values. Predefined cut-off values were used for Gleason score (≥ 8), PSA doubling time (PSADT) (6 months), and PSA response rate (90%), based on previous literature. All statistical analyses were two-sided, and *p*-values < 0.05 were considered statistically significant. Analyses were conducted using EZR version 1.55 (Saitama Medical Center, Jichi Medical University, Japan), a graphical user interface for R.

## Results

### Patient characteristics

A total of 67 patients with nmCRPC were enrolled in this study, including 32 in the APA group and 35 in the DARO group (Table 1). Prior to APA or DARO initiation, 8 patients (25.0%) in the APA group and 7 (20.0%) in the DARO group received flutamide. The median PSADT in the entire cohort was 4.4 months. In the APA group, 21 patients (65.6%) had a PSADT < 6 months, 5 patients (15.6%) had a PSADT of 6–10 months, and 6 patients (18.8%) had a PSADT > 10 months. In the DARO group, 16 patients (45.7%) had a PSADT < 6 months, 7 patients (20.0%) had a PSADT of 6–10 months, and 12 patients (34.3%) had a PSADT > 10 months. The median observation period was 26.6 months.

**Table 1:** The clinical characteristics of nmCRPC patients treated with APA or DARO

	total (*n* = 67)	APA (*n* = 32)	DARO (*n* = 35)	*p* value
PSA at diagnosis (ng/ml)	18.4 (3.0–1597.0)	21.8 (8.0–97.5)	14.8 (8.6–54.4)	0.572
Gleason score ≥ 8	40 (59.7)	17 (53.1)	23 (65.7)	0.328
History of local therapy	38 (56.7)	18 (56.3)	20 (57.1)	1.000
Radiation therapy	21 (31.3)	8 (25.0)	13 (37.1)	
Radical prostatectomy	17 (25.4)	10 (31.3)	7 (20.0)	
Previous use of flutamide	15 (22.4)	8 (25.0)	7 (20.0)	0.771
Time to CRPC (months)	61.8 (26.8–115.3)	54.8 (20.9–121.9)	65.8 (38.8–54.4)	0.697
PSADT (months)	4.4 (2.3–10.1)	3.8 (2.2–7.4)	6.7 (2.3–12.3)	0.248
PSA at APA or DARO initiation (ng/ml)	3.6 (2.2–6.5)	3.5 (2.4–6.5)	3.7 (1.9–6.1)	0.865
Age (years)	77.2 (7.7–84.0)	75.5 (69.7–82.3)	79.5 (73.3–84.2)	0.121
Observation periods (months)	26.6 (18.5–34.7)	34.8 (25.7–41.7)	21.5 (16.7–26.7)	< 0.001
Cancer specific death	13 (19.4)	7 (21.9)	6 (17.1)	0.760

*APA *Apalutamide, *DARO *Darolutamide,*PSA *Prostate-specific antigen, *CRPC *Castration-resistant prostate cancer, *PSADT *Prostate-specific antigen doubling time

^†^Continuous values are represented as median (IQR), and categorical values are shown as number of cases (% of each group)

^‡^nmCRPC, nonmetastatic castration-resistant prostate cancer

### Treatment duration and time to progression or discontinuation

**Fig. 1:**
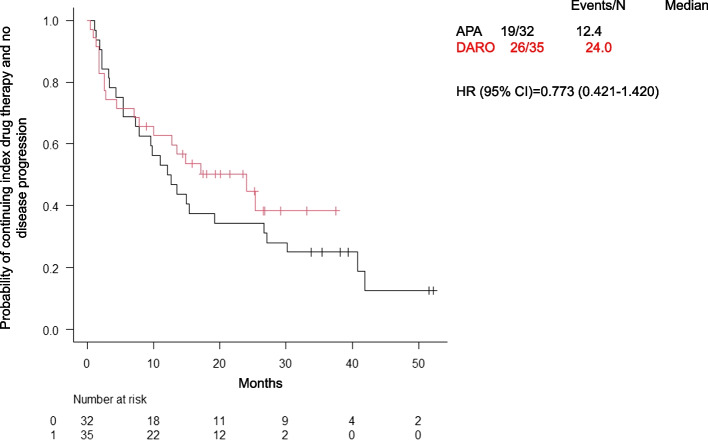
Time to APA or DARO discontinuation or progression to mCRPC. Abbreviations: APA, apalutamide; DARO, darolutamide; HR, hazard ratio; CI, confidence interval

The median time to treatment discontinuation or progression to mCRPC was longer in the DARO group (24.0 months) compared to the APA group (12.4 months), though this difference was not statistically significant (HR: 0.773; 95% CI: 0.421–1.420) (Fig. 1). No patients in either group continued treatment despite rising PSA levels. 20 patients (62.5%) in the APA group and 14 patients (40.0%) in the DARO group discontinued treatment prior to progression to mCRPC. In the APA group, treatment was discontinued due to TRAEs in 10 patients (31.3%), PSA progression without radiographic metastasis in 8 patients (25.0%), and other reasons in 2 patients (6.3%). In the DARO group, 6 patients 17.1%) discontinued due to TRAEs and 8 patients (22.9%) due to PSA progression.

### Progression to mCRPC

**Fig. 2:**
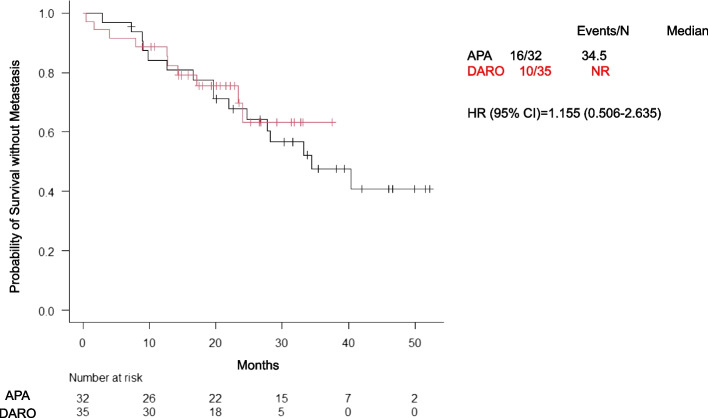
Time to progression to mCRPC between the APA and DARO groups. Abbreviations: mCRPC, metastatic castration-resistant prostate cancer; APA, apalutamide; DARO, darolutamide; HR, hazard ratio; CI, confidence interval; NR, not reached

**Fig. 3:**
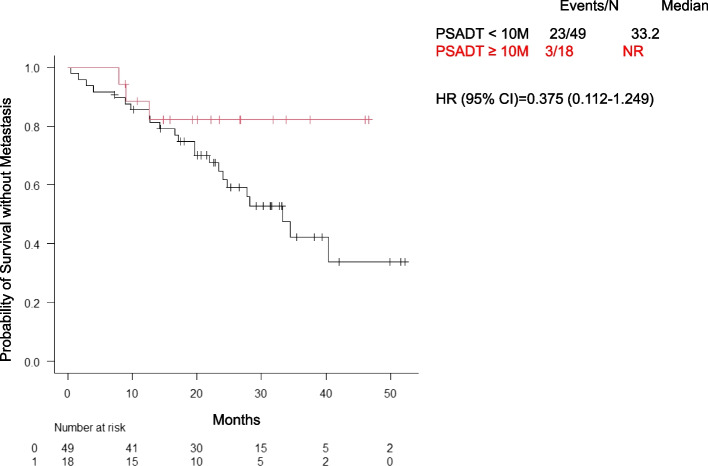
Time to progression to mCRPC by PSADT (< 10 vs ≥ 10 months). Abbreviations: mCRPC, metastatic castration-resistant prostate cancer; APA, apalutamide; DARO, darolutamide; HR, hazard ratio; CI, confidence interval; NR, not reached; PSADT, prostate-specific antigen doubling time

**Fig. 4:**
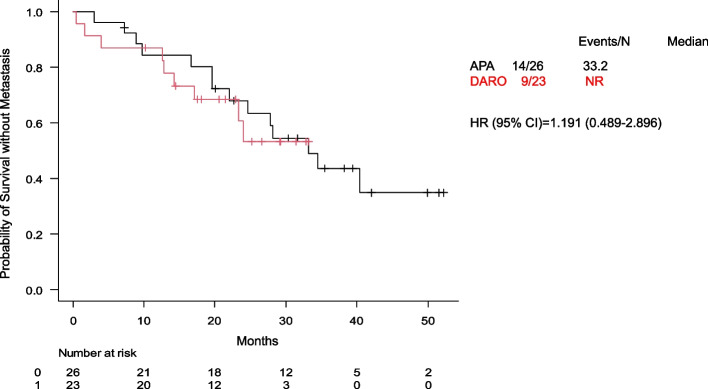
Time to mCRPC in APA vs DARO groups with PSADT < 10 months. Abbreviations: mCRPC, metastatic castration-resistant prostate cancer; APA, apalutamide; DARO, darolutamide; HR, hazard ratio; CI, confidence interval; NR, not reached; PSADT, prostate-specific antigen doubling time

The mCRPC progression rate was lower in the DARO group (28.6%, 10 patients) than in the APA group (50.0%, 16 patients). The median time to mCRPC was 34.5 months and was not reached in either group (HR: 1.155; 95% CI: 0.506–2.635) (Fig. 2). Kaplan–Meier analysis based on PSADT suggested that patients with PSADT ≥ 10 months had longer times to mCRPC than those with PSADT < 10 months (HR: 0.375; 95% CI: 0.112–1.249) (Fig. 3). However, in the subgroup with PSADT < 10 months, no significant difference was observed between the APA and DARO groups (Fig. 4).

### TRAEs

TRAEs occurred in 75.0% of patients in the APA group and 25.7% in the DARO group (*p* < 0.001) (Table 2). Rash was the most frequent TRAE, reported in 56.2% (18/32) of APA patients and 8.6% (3/35) of DARO patients. Fatigue was observed in 2 patients (6.2%) in the APA group and 1 patient (2.9%) in the DARO group. Treatment discontinuation due to TRAEs occurred in 10 patients (31.2%) in the APA group and 6 patients (17.1%) in the DARO group, indicating a clinically meaningful difference, although statistical significance was not reached. Dose reduction due to TRAEs was required in 2 patients (6.2%) in the APA group. In the DARO group, no patients continued treatment with a reduced dose. Grade ≥ 3 TRAEs were observed in 2 patients in the APA group (rash and fatigue) and in 3 patients in the DARO group (rash, pulmonary fibrosis, and fatigue).

**Table 2:** Treatment-related adverse events

	APA (*n* = 32)	DARO (*n* = 35)	*p* value
Any grade	24 (75.0)	9 (25.7)	< 0.001
Rash	18 (56.2)	3 (8.6)	< 0.001
Pulmonary fibrosis	0	1 (2.9)	ns
Fatigue	2 (6.2)	1 (2.9)	ns
Anemia	0	1 (2.9)	ns
Diarrhea	0	1 (2.9)	ns
Hot flush	0	1 (2.9)	ns
Arthralgia	0	1 (2.9)	ns
Nausea	1 (3.1)	0	ns
Hypertension	1 (3.1)	0	ns
Alanine aminotransferase increased	1 (3.1)	0	ns
Fracture	1 (3.1)	0	ns
Mucositis oral	1 (3.1)	0	ns
Peripheral sensory neuropathy	1 (3.1)	0	ns
			ns
Grade ≥ 3	2 (6.2)	3 (8.6)	1.000
Rash	1 (3.1)	1 (2.9)	ns
Pulmonary fibrosis	0	1 (2.9)	ns
Fatigue	1 (3.1)	1 (2.9)	ns

^†^Categorical values are shown as number of cases (%)

^‡^The category of"rash"includes the following MedDRA terms: dermatitis, erythema, rash, macular rash, maculopapular rash, papular rash, and pustular rash

^§^APA, apalutamide; DARO, darolutamide; ns, not significant

### PSA response

**Fig. 5:**
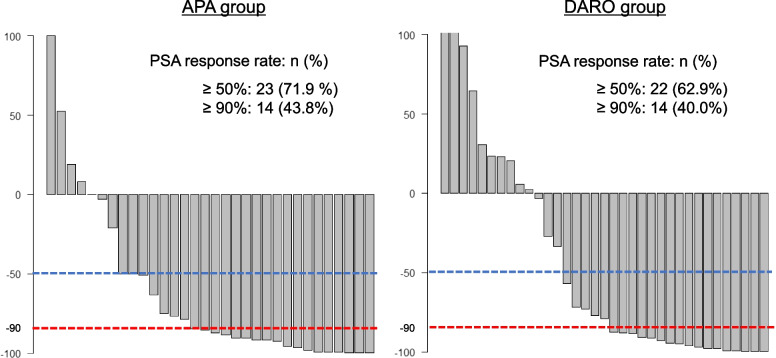
PSA response rates in the APA and DARO groups. Abbreviations: PSA, prostate-specific antigen; APA, apalutamide; DARO, darolutamide

PSA response rates were similar between groups (*p* = 0.208). A PSA reduction ≥ 90% was observed in 14 patients (43.8%) in the APA group and 14 (40.0%) in the DARO group (Fig. 5).

### Patterns of metastasis and sequential therapy

Among patients who progressed to mCRPC, the most common sites of metastasis in both groups were lymph nodes and bones (Table 3).

**Table 3:** Location of metastasis after progression to mCRPC

	APA (*n* = 32)	DARO (*n* = 35)
Lymph node	13 (40.6)	7 (20.0)
Bone	6 (18.8)	4 (11.4)
Lung	1 (3.1)	3 (8.6)
Liver	1 (3.1)	1 (2.9)

^†^Categorical values are shown as number of cases (%)

^‡^mCRPC, metastatic castration-resistant prostate cancer; APA, apalutamide; DARO, darolutamide

Sankey diagrams (Additional file 1 and 2) illustrate the sequential therapy following mCRPC progression. In both groups, abiraterone acetate (ABI) was the most frequently used first-line agent in the mCRPC setting, used in 8 patients (25.0%) in the APA group and 4 (11.4%) in the DARO group. Only a few patients were able to receive third-line therapy, with 31.3% (5/16) in the APA group and 20.0% (2/10) in the DARO group.

### Multivariate analysis of predictors for mCRPC progression

Multivariate Cox regression analysis identified two independent predictors of progression to mCRPC: PSA level ≥ 3.6 ng/mL at APA or DARO initiation (HR: 3.18, *p* < 0.001) and PSA response rate < 90% (HR: 6.03, *p* < 0.001) (Table 4).

**Table 4:** Multivariate analyses of several parameters as predictors of progression to mCRPC

Variables	Initial model			Final model		
	HR	95% CI	*p* value	HR	95% CI	*p* value
Gleason score (< 8 vs ≥ 8)	0.71	0.28–1.79	0.467			
History of local therapy	1.26	0.43–3.71	0.671			
Time to CRPC (< 61 vs ≥ 61) (months)	1.03	0.34–3.09	0.958			
PSADT (< 6 vs ≥ 6) (months)	2.22	0.92–5.38	0.078	2.27	0.94–5.48	0.067
Previous use of flutamide	0.90	0.36–2.24	0.820			
PSA at APA or DARO initiation (< 3.6 vs ≥ 3.6) (ng/ml)	2.81	1.13–7.03	0.027	3.18	1.37–7.38	< 0.001
Age (< 77 vs ≥ 77) (years)	1.27	0.56–2.90	0.569			
PSA response rate (< 90 vs ≥ 90) (%)	6.74	2.27–20.0	< 0.001	6.03	2.11–17.2	< 0.001

*CRPC* Castration-resistant prostate cancer, *PSADT* Prostate-specific antigen doubling time, *APA* Apalutamide, *DARO* Darolutamide, *PSA* Prostate-specific antigen

^†^mCRPC, metastatic castration-resistant prostate cancer

## Discussion

Real-world data on ARPIs in Japanese patients with nmCRPC have been reported in several studies, and the number of reports has increased since 2024 (Additional file 3). These studies have examined the efficacy and treatment initiation timing, compared the efficacy and safety of ARPIs, and analyzed the prognostic factors [5–12]. In particular, APA is associated with a high incidence of rash [7, 8], which may affect treatment continuation and quality of life. Additionally, PSA levels at initiation and PSADT are associated with prognosis in nmCRPC [5–7, 9–12], indicating the need for further investigation of their role in predicting disease progression. Our study adds to the literature by comparing the efficacy and safety of APA and DARO, identifying predictors of progression to mCRPC, and assessing the time to treatment discontinuation or progression to mCRPC for the first time in a real-world Japanese cohort. Furthermore, we investigated sequential therapy after progression to mCRPC.

In this study, among patients who met the clinical trial criteria of PSADT < 10 months, efficacy outcomes such as time to mCRPC progression were similar between the APA and DARO groups. However, the treatment discontinuation rate due to TRAEs was lower in the DARO group (17.1% vs. 31.2%), indicating better tolerability. These findings are consistent with the results of a large-scale retrospective cohort study, the DEAR study, reported in 2024 [13]. The DEAR study demonstrated that compared to APA and enzalutamide (ENZ), DARO was associated with significantly lower treatment discontinuation and mCRPC progression rates, as well as a longer treatment duration. These trends were also observed in the real-world clinical data of the Japanese patients in our study. Because DARO has low blood–brain barrier permeability and fewer drug interactions, it is associated with fewer TRAEs and better treatment adherence, even in elderly patients. DARO may be a promising treatment option for the long-term management of nmCRPC, particularly in terms of safety and treatment continuity.

More than half of the patients in the APA group (56%, 18/32 cases) experienced rash, showing a higher incidence than that reported in the SPARTAN trial (23.8%) [1]. The incidence of APA-induced rash was higher in Japanese populations than in Western populations [14, 15]. In the analysis of Japanese patients from the SPARTAN and TITAN trials, the incidence of rash was reported to be 51.5%, nearly twice as high as that observed in the global population [15]. Additionally, in real-world clinical data, Hara et al. reported a relatively lower rash incidence of approximately 27.8% [8], whereas Fujiwara et al. reported an incidence of 50.0%, which aligns with our findings [7]. Conversely, a German cohort study reported a lower rash incidence of 16.1% [16], suggesting a lower prevalence in non-Japanese populations. Possible reasons for the higher incidence of rash in Japanese patients include higher plasma concentrations of APA due to lower body weight compared to that of Western populations and differences in specific HLA genetic polymorphisms, which may influence skin reaction sensitivity to APA [14]. Given these findings, appropriate management, such as dose interruption or dose reduction, is crucial while administering APA to Japanese patients to mitigate the risk of rash.

In both groups, rash was the primary reason for treatment discontinuation due to TRAEs. In particular, in the APA group, 9 of 10 cases of TRAE-related discontinuation were attributed to rash, whereas in the DARO group, 3 out of 6 cases were also due to rash. Notably, rash associated with DARO was not reported as a frequent TRAE (2.9%) in the ARAMIS trial [3]. In real-world reports, there have been no documented cases of rash associated with DARO [7, 17]. Further research is needed to determine whether the DARO-related rash observed in this study indicates that, similar to APA, DARO also causes rash more frequently in Japanese patients or whether it is influenced by specific patient characteristics other than race.

We also investigated the predictors of progression to mCRPC. Previous studies have identified a high GS, short PSADT, high PSA levels at treatment initiation, a history of local therapy, and a high PSA response rate as predictors of MFS in nmCRPC [9–12, 18–24]. Similarly, our study found that high PSA levels at APA or DARO initiation (≥ 3.6 ng/mL) and a high PSA response rate (≥ 90%) were independent predictors of progression to mCRPC, supporting these findings. In patients with nmCRPC, high PSA levels are associated with the detection of metastases on prostate-specific membrane antigen ligand positron emission tomography (PSMA-PET) [25, 26]. Fendler et al. reported that, among patients diagnosed with nmCRPC by conventional imaging (CT, magnetic resonance imaging, and bone scintigraphy), PSMA-PET detected distant metastases in 55% of cases, and PSA ≥ 5.5 ng/mL was identified as a predictive factor [24]. In this study as well, patients with high PSA levels may be among those treated for nmCRPC who had micrometastases detectable by PSMA-PET, potentially leading to early progression to mCRPC. Additionally, multiple post hoc analyses of clinical trials, including the SPARTAN, IMMGEN, and TITAN trials, have demonstrated that achieving a PSA response rate ≥ 90% is associated with prolonged MFS and OS in both mCSPC and nmCRPC [20, 21, 23, 24]. Although similar findings have been reported in real-world studies of mCSPC [27], there have been no reports on nmCRPC. Our findings suggest that achieving a PSA response rate of ≥ 90% may also improve prognosis in real-world nmCRPC settings.

This study has several limitations. First, it was a retrospective single-center study with a limited sample size, and treatment allocation was not randomized, which may have introduced potential bias. Second, 26.9% of patients had a PSA doubling time (PSADT) > 10 months, although ARPIs are typically recommended for PSADT ≤ 10 months by Japanese prostate cancer clinical guideline 2023. Third, some patients had received flutamide before APA or DARO initiation, which may differ from standard trial protocols. Finally, we defined time to mCRPC progression from the start of APA or DARO treatment, rather than from the diagnosis of nmCRPC, as in most clinical trials. Despite these limitations, the study provides meaningful insights into real-world management of nmCRPC in Japanese patients.

## Conclusions

DARO was associated with lower toxicity compared to APA in Japanese patients with nmCRPC. Rash was more prevalent with APA. Elevated baseline PSA and suboptimal PSA response were associated with progression.

## Supplementary Information

Additional file 1. Sequential therapy after progression to mCRPC in the APA group. Abbreviations: mCRPC, metastatic castration-resistant prostate cancer; APA, n, number of cases; apalutamide; ABI, abiraterone acetate; DOC, docetaxel; ENZ, enzalutamide; CBZ, cabazitaxel; Ra-223, radium-223; BSC, best supportive care.

Additional file 2. Sequential therapy after progression to mCRPC in the DARO group. Abbreviations: mCRPC, metastatic castration-resistant prostate cancer; DARO, darolutamide; n, number of cases; ABI, abiraterone acetate; BSC, best supportive care; DOC, docetaxel, docetaxel; ENZ, enzalutamide; CBZ, cabazitaxel; Ra-223, radium-223; Vintage, vintage nonsteroidal antiandrogen agent.

Additional file 3. Studies on ARPIs in Japanese patients with nmCRPC. Abbreviations: n, number of cases; ARPI, androgen receptor pathway inhibitors; nmCRPC, nonmetastatic castration-resistant prostate cancer; MFS, metastasis-free survival; PSA, prostate-specific antigen; TRAEs, treatment-related adverse events; APA, apalutamide; DARO, darolutamide; ENZ, enzalutamide; ABI, abirateron acetate; OS, overall survival; PSADT, prostate-specific antigen doubling time; PSA at initiation, prostate-specific antigen level at treatment initiation; CRPC, castration-resistant prostate cancer; NR, not reached.

## Data Availability

The datasets used and/or analyzed during the current study are available from the corresponding author on reasonable request.

## Abbreviations

nmCRPC: Non-metastatic castration-resistant prostate cancer
mCRPC: Metastatic castration-resistant prostate cancer
APA: Apalutamide
DARO: Darolutamide
ENZ: Enzalutamide
ABI: Abirateron acetate
DTX: Docetaxel
MFS: Metastasis-free survival
OS: Overall survival
PSA: Prostate-specific antigen
PSADT: Prostate-specific antigen doubling time
ADT: Androgen deprivation therapy
CAB: Combined androgen blockade
TRAEs: Treatment-related adverse events
ARPI: Androgen receptor pathway inhibitor
CTCAE: Common Terminology Criteria for Adverse Events
GS: Gleason score
PSMA-PET: Prostate specific membrane antigen ligand positron emission tomography
