# Supplementary figures and images for: Comparative real-world study of apalutamide and darolutamide in Japanese patients with non-metastatic castration-resistant prostate cancer
Source: BMC Urol. 2025 Sep 26;25:235. doi: 10.1186/s12894-025-01919-z (PMC12465178; doi:10.1186/s12894-025-01919-z)

## Slide 1
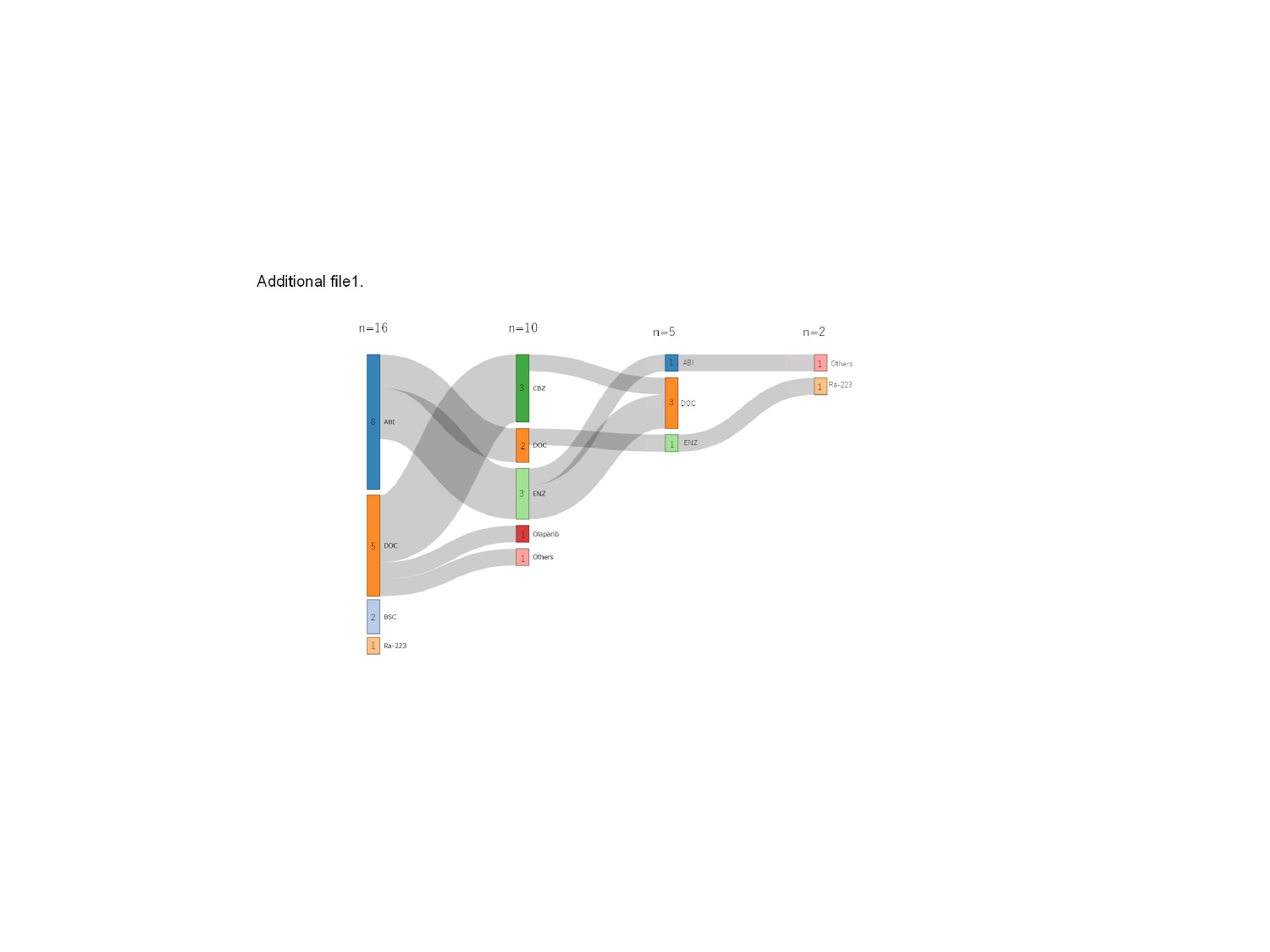

Supplement: Supplementary file 1 — Additional file 1. Sequential therapy after progression to mCRPC in the APA group. Abbreviations: mCRPC, metastatic castration-resistant prostate cancer; APA, n, number of cases; apalutamide; ABI, abiraterone acetate; DOC, docetaxel; ENZ, enzalutamide; CBZ, cabazitaxel; Ra-223, radium-223; BSC, best supportive care. [file 12894_2025_1919_MOESM1_ESM.pptx]

## Slide 1
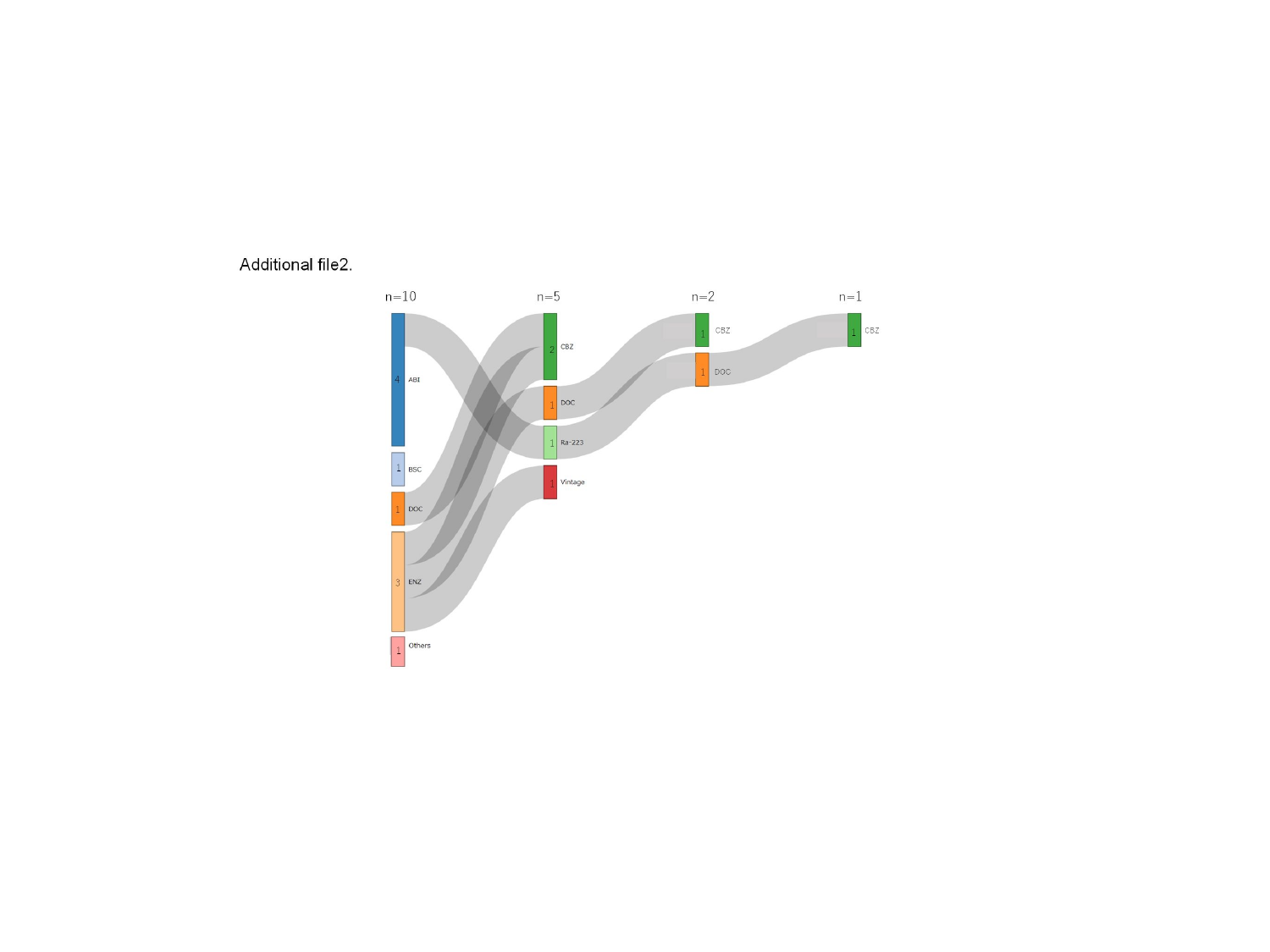

Supplement: Supplementary file 2 — Additional file 2. Sequential therapy after progression to mCRPC in the DARO group. Abbreviations: mCRPC, metastatic castration-resistant prostate cancer; DARO, darolutamide; n, number of cases; ABI, abiraterone acetate; BSC, best supportive care; DOC, docetaxel, docetaxel; ENZ, enzalutamide; CBZ, cabazitaxel; Ra-223, radium-223; Vintage, vintage nonsteroidal antiandrogen agent. [file 12894_2025_1919_MOESM2_ESM.pptx]
